# Supplementary material for: Evaluation of Immunogenicity and Safety of Vero Cell-Derived Inactivated COVID-19 Vaccine in Older Patients with Hypertension and Diabetes Mellitus
Source: Vaccines (Basel). 2022 Jun 25;10(7):1020. doi: 10.3390/vaccines10071020 (PMC9315836; doi:10.3390/vaccines10071020)
Supplement: Supplementary file 1 [file vaccines-10-01020-s001.zip › Supplementary-Method.pdf]

Method for SARS-CoV-2 neutralizing assay

The neutralization antibody assays were performed at the National Institute for Viral Disease Control and Prevention, China CDC, Beijing 1. Serum samples were measured for neutralization capacity testing using infectious SARS-CoV-2 virus (strain 19nCoVCDC-Tan-Strain04 [QD01]) by the 50% cell culture infectious dose. Serum was successively diluted 1:4 to the required concentration by a 2-fold series, and an equal volume of challenge virus solution was added. After neutralization in a 37 °C incubator for 2 h, a 1.0~2.5×105/mL cell suspension was added to the wells (0.1 mL per well) and cultured in a CO2 incubator at 37 °C for 4 days. Titers expressed as the reciprocal of the highest dilution protecting 50% cell from virus challenge. Convalescent sera is included as an internal positive control in every assay.

References

1. Xia S, Zhang Y, Wang Y, et al. Safety and immunogenicity of an inactivated SARS-CoV-2 vaccine, BBIBP-CorV: a randomised, double-blind, placebo-controlled, phase 1/2 trial. Lancet Infect Dis 2021; 21(1): 39-51.
