# Supplementary material for: Evaluation of Immunogenicity and Safety of Vero Cell-Derived Inactivated COVID-19 Vaccine in Older Patients with Hypertension and Diabetes Mellitus
Source: Vaccines (Basel). 2022 Jun 25;10(7):1020. doi: 10.3390/vaccines10071020 (PMC9315836; doi:10.3390/vaccines10071020)
Supplement: Supplementary file 1 [file vaccines-10-01020-s001.zip › SupplementaryTable1-5_0602.pdf]

**Table S1. Neutralizing antibodies to live SARS-Cov-2**

| Variable | Geometric mean titers (95% CI) | *p* value* |
|---|---|---|
| Comorbidity | | |
| Healthy population | 74.86(69.49,80.64) | - |
| Hypertension group | 73.41(67.24,80.14) | 0.567 |
| Diabetes group | 69.93(63.59,76.89) | 0.095 |
| Combined disease group | 73.84(66.70,81.75) | 0.567 |
| Age | | 0.114 |
| 60-69 years older | 75.33(71.52,79.35) | |
| 70 years and older | 68.35(62.96,74.19) | |
| Gender | | <0.001 |
| Male | 63.04(59.14,67.20) | |
| Female | 83.35(78.57,88.42) | |
| Age * Gender | | 0.105 |

*Analyzed by Generalized Linear Model

**Table S2-1 Adverse events related to the study vaccine 0-21 days after two doses of vaccine in the male**

| Adverse events | Hypertension group (157 assigned) | | | Diabetes group (137 assigned) | | | Combined disease group (120 assigned) | | | Healthy population (256 assigned) | | | | | |
|---|---|---|---|---|---|---|---|---|---|---|---|---|---|---|---|
| | Case times | No. of cases | Incidence rate | Case times | No. of cases | Incidence rate | Case times | No. of cases | Incidence rate | Case times | No. of cases | Incidence rate | Statistical method | Statistic | *P** |
| Total | 39 | 25 | 15.92 | 77 | 29 | 21.17 | 54 | 20 | 16.67 | 80 | 38 | 14.84 | Chi-Square Test | 2.666 | 0.4461 |
| Solicited event | 22 | 17 | 10.83 | 43 | 21 | 15.33 | 20 | 8 | 6.67 | 25 | 14 | 5.47 | Chi-Square Test | 12.088 | 0.0071 |
| Local adverse events | 8 | 8 | 5.10 | 10 | 9 | 6.57 | 2 | 2 | 1.67 | 7 | 6 | 2.34 | Fisher's Exact Test | - | 0.0844 |
| Soreness | 6 | 6 | 3.82 | 7 | 6 | 4.38 | 1 | 1 | 0.83 | 7 | 6 | 2.34 | Fisher's Exact Test | - | 0.2774 |
| Pruritus | 1 | 1 | 0.64 | 1 | 1 | 0.73 | 1 | 1 | 0.83 | 0 | 0 | 0.00 | Fisher's Exact Test | - | 0.3197 |
| Systemic adverse events | 14 | 10 | 6.37 | 33 | 15 | 10.95 | 18 | 8 | 6.67 | 18 | 10 | 3.91 | Chi-Square Test | 7.385 | 0.0606 |
| Dizziness | 6 | 5 | 3.18 | 12 | 9 | 6.57 | 3 | 3 | 2.50 | 8 | 6 | 2.34 | Fisher's Exact Test | - | 0.1907 |
| Fatigue/lethargy | 1 | 1 | 0.64 | 4 | 4 | 2.92 | 3 | 3 | 2.50 | 3 | 3 | 1.17 | Fisher's Exact Test | - | 0.3459 |
| Headache | 2 | 2 | 1.27 | 5 | 5 | 3.65 | 2 | 2 | 1.67 | 0 | 0 | 0.00 | Fisher's Exact Test | - | 0.0102 |
| Cough | 1 | 1 | 0.64 | 1 | 1 | 0.73 | 3 | 3 | 2.50 | 2 | 2 | 0.78 | Fisher's Exact Test | - | 0.5097 |
| Chest tightness | 1 | 1 | 0.64 | 4 | 3 | 2.19 | 1 | 1 | 0.83 | 0 | 0 | 0.00 | Fisher's Exact Test | - | 0.0580 |
| Nausea | 1 | 1 | 0.64 | 3 | 3 | 2.19 | 1 | 1 | 0.83 | 0 | 0 | 0.00 | Fisher's Exact Test | - | 0.0580 |
| Arthralgia | 1 | 1 | 0.64 | 0 | 0 | 0.00 | 0 | 0 | 0.00 | 2 | 2 | 0.78 | Fisher's Exact Test | - | 0.8897 |
| Muscle pain | 1 | 1 | 0.64 | 0 | 0 | 0.00 | 0 | 0 | 0.00 | 2 | 2 | 0.78 | Fisher's Exact Test | - | 0.8897 |
| Anorexia | 0 | 0 | 0.00 | 1 | 1 | 0.73 | 0 | 0 | 0.00 | 1 | 1 | 0.39 | Fisher's Exact Test | - | 0.8207 |
| Unsolicited event | 17 | 14 | 8.92 | 34 | 13 | 9.49 | 34 | 15 | 12.50 | 55 | 30 | 11.72 | Chi-Square Test | 1.411 | 0.7031 |
| Musculoskeletal and connective tissue disorders | 2 | 2 | 1.27 | 9 | 4 | 2.92 | 13 | 4 | 3.33 | 14 | 6 | 2.34 | Fisher's Exact Test | - | 0.6855 |

| | | | | | | | | | | | | | | | |
|---|---|---|---|---|---|---|---|---|---|---|---|---|---|---|---|
| Gastrointestinal diseases | 0 | 0 | 0.00 | 7 | 4 | 2.92 | 3 | 2 | 1.67 | 16 | 9 | 3.52 | Fisher's Exact Test | - | 0.0661 |
| Systemic disease and administration site reactions | 2 | 2 | 1.27 | 7 | 6 | 4.38 | 5 | 5 | 4.17 | 5 | 3 | 1.17 | Fisher's Exact Test | - | 0.0868 |
| Respiratory system, chest and mediastinal diseases | 5 | 4 | 2.55 | 3 | 2 | 1.46 | 1 | 1 | 0.83 | 4 | 3 | 1.17 | Fisher's Exact Test | - | 0.6911 |
| Neurological diseases | 3 | 3 | 1.91 | 3 | 2 | 1.46 | 2 | 2 | 1.67 | 4 | 4 | 1.56 | Fisher's Exact Test | - | 1.0000 |
| Metabolic and nutritional | 1 | 1 | 0.64 | 2 | 2 | 1.46 | 4 | 2 | 1.67 | 0 | 0 | 0.00 | Fisher's Exact Test | - | 0.1020 |
| Kidney and urinary system | 0 | 0 | 0.00 | 1 | 1 | 0.73 | 0 | 0 | 0.00 | 6 | 4 | 1.56 | Fisher's Exact Test | - | 0.3245 |
| Skin and subcutaneous tissue | 3 | 3 | 1.91 | 0 | 0 | 0.00 | 0 | 0 | 0.00 | 1 | 1 | 0.39 | Fisher's Exact Test | - | 0.1407 |
| Injuries, poisoning and operation complications | 1 | 1 | 0.64 | 0 | 0 | 0.00 | 0 | 0 | 0.00 | 2 | 1 | 0.39 | Fisher's Exact Test | - | 1.0000 |
| Reproductive system and breast diseases | 0 | 0 | 0.00 | 0 | 0 | 0.00 | 0 | 0 | 0.00 | 2 | 1 | 0.39 | Fisher's Exact Test | - | 1.0000 |
| Heart diseases | 0 | 0 | 0.00 | 0 | 0 | 0.00 | 0 | 0 | 0.00 | 1 | 1 | 0.39 | Fisher's Exact Test | - | 1.0000 |

*Kruskal-Wallis test

## Table S2-2 Adverse events related to the study vaccine 0-21 days after two doses of vaccine in the female

| Adverse events | Hypertension group (170 assigned) | | | Diabetes group (192 assigned) | | | Combined disease group (176 assigned) | | | Healthy population (224 assigned) | | | | | |
|---|---|---|---|---|---|---|---|---|---|---|---|---|---|---|---|
| | Case times | No. of cases | Incidence rate | Case times | No. of cases | Incidence rate | Case times | No. of cases | Incidence rate | Case times | No. of cases | Incidence rate | Statistical method | Statistic | *P** |
| Total | 60 | 35 | 20.59 | 67 | 39 | 20.31 | 78 | 33 | 18.75 | 85 | 43 | 19.20 | Chi-Square Test | 0.267 | 0.9662 |
| Solicited event | 27 | 22 | 12.94 | 39 | 26 | 13.54 | 49 | 24 | 13.64 | 45 | 27 | 12.05 | Chi-Square Test | 0.291 | 0.9617 |
| Local adverse events | 5 | 5 | 2.94 | 11 | 9 | 4.69 | 13 | 10 | 5.68 | 17 | 14 | 6.25 | Chi-Square Test | 2.471 | 0.4805 |
| Soreness | 5 | 5 | 2.94 | 8 | 7 | 3.65 | 8 | 7 | 3.98 | 16 | 13 | 5.80 | Chi-Square Test | 2.270 | 0.5184 |
| Pruritus | 0 | 0 | 0.00 | 0 | 0 | 0.00 | 1 | 1 | 0.57 | 1 | 1 | 0.45 | Fisher's Exact | - | 0.8517 |
| Systemic adverse events | 22 | 18 | 10.59 | 28 | 20 | 10.42 | 36 | 15 | 8.52 | 28 | 16 | 7.14 | Chi-Square Test | 1.973 | 0.5781 |
| Dizziness | 9 | 8 | 4.71 | 13 | 10 | 5.21 | 5 | 4 | 2.27 | 7 | 6 | 2.68 | Chi-Square Test | 3.392 | 0.3351 |
| Fatigue/lethargy | 7 | 6 | 3.53 | 5 | 5 | 2.60 | 7 | 5 | 2.84 | 6 | 6 | 2.68 | Fisher's Exact | - | 0.9551 |
| Headache | 1 | 1 | 0.59 | 2 | 2 | 1.04 | 6 | 5 | 2.84 | 3 | 3 | 1.34 | Fisher's Exact | - | 0.3842 |
| Non-injection site pruritus | 1 | 1 | 0.59 | 0 | 0 | 0.00 | 1 | 1 | 0.57 | 4 | 2 | 0.89 | Fisher's Exact | - | 0.7886 |
| Muscle pain | 1 | 1 | 0.59 | 2 | 2 | 1.04 | 0 | 0 | 0.00 | 3 | 2 | 0.89 | Fisher's Exact | - | 0.7310 |
| Nausea | 0 | 0 | 0.00 | 1 | 1 | 0.52 | 3 | 3 | 1.70 | 1 | 1 | 0.45 | Fisher's Exact | - | 0.2929 |
| Chest tightness | 0 | 0 | 0.00 | 1 | 1 | 0.52 | 3 | 3 | 1.70 | 1 | 1 | 0.45 | Fisher's Exact | - | 0.2929 |
| Cough | 1 | 1 | 0.59 | 1 | 1 | 0.52 | 2 | 2 | 1.14 | 0 | 0 | 0.00 | Fisher's Exact | - | 0.4191 |
| Vomiting | 1 | 1 | 0.59 | 0 | 0 | 0.00 | 0 | 0 | 0.00 | 1 | 1 | 0.45 | Fisher's Exact | - | 0.7157 |
| Constipation | 0 | 0 | 0.00 | 0 | 0 | 0.00 | 0 | 0 | 0.00 | 1 | 1 | 0.45 | Fisher's Exact | - | 1.0000 |
| Anorexia | 0 | 0 | 0.00 | 0 | 0 | 0.00 | 0 | 0 | 0.00 | 1 | 1 | 0.45 | Fisher's Exact | - | 1.0000 |

| | | | | | | | | | | | | | | |
|---|---|---|---|---|---|---|---|---|---|---|---|---|---|---|
| Unsolicited event | 33 | 21 | 12.35 | 28 | 17 | 8.85 | 29 | 16 | 9.09 | 40 | 23 | 10.27 | Chi-Square Test | 1.482 | 0.6864 |
| Systemic disease and administration site reactions | 5 | 4 | 2.35 | 9 | 4 | 2.08 | 8 | 6 | 3.41 | 5 | 3 | 1.34 | Fisher's Exact Test | - | 0.5695 |
| Gastrointestinal diseases | 2 | 2 | 1.18 | 4 | 3 | 1.56 | 4 | 2 | 1.14 | 7 | 3 | 1.34 | Fisher's Exact | - | 1.0000 |
| Musculoskeletal and connective tissue disorders | 1 | 1 | 0.59 | 2 | 2 | 1.04 | 7 | 5 | 2.84 | 5 | 4 | 1.79 | Fisher's Exact Test | - | 0.3713 |
| Neurological diseases | 2 | 2 | 1.18 | 3 | 3 | 1.56 | 3 | 2 | 1.14 | 5 | 4 | 1.79 | Fisher's Exact | - | 0.9538 |
| Respiratory system, chest and mediastinal diseases | 4 | 4 | 2.35 | 1 | 1 | 0.52 | 3 | 3 | 1.70 | 4 | 3 | 1.34 | Fisher's Exact Test | - | 0.4819 |
| Infections and infectious | 3 | 3 | 1.76 | 3 | 3 | 1.56 | 2 | 2 | 1.14 | 3 | 3 | 1.34 | Fisher's Exact | - | 0.9765 |
| Skin and subcutaneous tissue diseases | 2 | 1 | 0.59 | 1 | 1 | 0.52 | 0 | 0 | 0.00 | 3 | 3 | 1.34 | Fisher's Exact Test | - | 0.5063 |
| Injuries, poisoning and operation complications | 1 | 1 | 0.59 | 0 | 0 | 0.00 | 1 | 1 | 0.57 | 3 | 2 | 0.89 | Fisher's Exact Test | - | 0.7886 |
| Eye diseases | 2 | 1 | 0.59 | 0 | 0 | 0.00 | 0 | 0 | 0.00 | 3 | 2 | 0.89 | Fisher's Exact | - | 0.5028 |
| Mental illness | 0 | 0 | 0.00 | 0 | 0 | 0.00 | 0 | 0 | 0.00 | 1 | 1 | 0.45 | Fisher's Exact | - | 1.0000 |
| Blood and lymphatic system diseases | 0 | 0 | 0.00 | 0 | 0 | 0.00 | 0 | 0 | 0.00 | 1 | 1 | 0.45 | Fisher's Exact Test | - | 1.0000 |

*Kruskal-Wallis test

## Table S3-1 Severity of adverse events 0-21 days after two doses of vaccine in the male

| Adverse events | Hypertension group (157 assigned) | | | Diabetes group (137 assigned) | | | Combined disease group (120 assigned) | | | Healthy population (256 assigned) | | | |
|---|---|---|---|---|---|---|---|---|---|---|---|---|---|
| | Level 1 | Level 2 | Level 3 | Level 1 | Level 2 | Level 3 | Level 1 | Level 2 | Level 3 | Level 1 | Level 2 | Level 3 | *P** |
| Total | 21(13.38) | 3(1.91) | 1(0.64) | 19(13.87) | 6(4.38) | 4(2.92) | 11(9.17) | 6(5.00) | 3(2.50) | 22(8.59) | 13(5.08) | 3(1.17) | 0.4399 |
| Solicitation event | 15(9.55) | 1(0.64) | 1(0.64) | 16(11.68) | 4(2.92) | 1(0.73) | 5(4.17) | 2(1.67) | 1(0.83) | 12(4.69) | 2(0.78) | 0(0.00) | 0.0070 |
| Local adverse events | 8(5.10) | 0(0.00) | 0(0.00) | 9(6.57) | 0(0.00) | 0(0.00) | 2(1.67) | 0(0.00) | 0(0.00) | 6(2.34) | 0(0.00) | 0(0.00) | 0.0831 |
| Soreness | 6(3.82) | 0(0.00) | 0(0.00) | 6(4.38) | 0(0.00) | 0(0.00) | 1(0.83) | 0(0.00) | 0(0.00) | 6(2.34) | 0(0.00) | 0(0.00) | 0.2952 |
| Pruritus | 1(0.64) | 0(0.00) | 0(0.00) | 1(0.73) | 0(0.00) | 0(0.00) | 1(0.83) | 0(0.00) | 0(0.00) | 0(0.00) | 0(0.00) | 0(0.00) | 0.5893 |
| Swelling | 0(0.00) | 0(0.00) | 0(0.00) | 2(1.46) | 0(0.00) | 0(0.00) | 0(0.00) | 0(0.00) | 0(0.00) | 0(0.00) | 0(0.00) | 0(0.00) | 0.0505 |
| Rash | 1(0.64) | 0(0.00) | 0(0.00) | 0(0.00) | 0(0.00) | 0(0.00) | 0(0.00) | 0(0.00) | 0(0.00) | 0(0.00) | 0(0.00) | 0(0.00) | 0.3522 |
| Systemic adverse events | 9(5.73) | 3(1.91) | 1(0.64) | 20(14.60) | 6(4.38) | 3(2.19) | 11(9.17) | 3(2.50) | 4(3.33) | 14(5.47) | 2(0.78) | 0(0.00) | <0.0001 |
| Dizziness | 4(2.55) | 1(0.64) | 0(0.00) | 8(5.84) | 1(0.73) | 0(0.00) | 2(1.67) | 1(0.83) | 0(0.00) | 6(2.34) | 0(0.00) | 0(0.00) | 0.1504 |
| Fatigue/lethargy | 1(0.64) | 0(0.00) | 0(0.00) | 2(1.46) | 1(0.73) | 1(0.73) | 1(0.83) | 1(0.83) | 1(0.83) | 3(1.17) | 0(0.00) | 0(0.00) | 0.3466 |
| Headache | 2(1.27) | 0(0.00) | 0(0.00) | 3(2.19) | 1(0.73) | 1(0.73) | 2(1.67) | 0(0.00) | 0(0.00) | 0(0.00) | 0(0.00) | 0(0.00) | 0.0279 |
| Cough | 1(0.64) | 0(0.00) | 0(0.00) | 0(0.00) | 1(0.73) | 0(0.00) | 2(1.67) | 0(0.00) | 1(0.83) | 1(0.39) | 1(0.39) | 0(0.00) | 0.3901 |
| Nausea | 0(0.00) | 1(0.64) | 0(0.00) | 2(1.46) | 0(0.00) | 1(0.73) | 1(0.83) | 0(0.00) | 0(0.00) | 0(0.00) | 0(0.00) | 0(0.00) | 0.1214 |
| Chest tightness | 1(0.64) | 0(0.00) | 0(0.00) | 3(2.19) | 0(0.00) | 0(0.00) | 1(0.83) | 0(0.00) | 0(0.00) | 0(0.00) | 0(0.00) | 0(0.00) | 0.1213 |
| Non-injection site | 0(0.00) | 0(0.00) | 0(0.00) | 1(0.73) | 0(0.00) | 0(0.00) | 1(0.83) | 1(0.83) | 0(0.00) | 0(0.00) | 0(0.00) | 0(0.00) | 0.1069 |
| Arthralgia | 0(0.00) | 1(0.64) | 0(0.00) | 0(0.00) | 0(0.00) | 0(0.00) | 0(0.00) | 0(0.00) | 0(0.00) | 1(0.39) | 1(0.39) | 0(0.00) | 0.5898 |
| Muscle pain | 0(0.00) | 0(0.00) | 1(0.64) | 0(0.00) | 0(0.00) | 0(0.00) | 0(0.00) | 0(0.00) | 0(0.00) | 2(0.78) | 0(0.00) | 0(0.00) | 0.5900 |
| Diarrhea | 0(0.00) | 0(0.00) | 0(0.00) | 0(0.00) | 1(0.73) | 0(0.00) | 0(0.00) | 0(0.00) | 1(0.83) | 0(0.00) | 0(0.00) | 0(0.00) | 0.3557 |
| Anorexia | 0(0.00) | 0(0.00) | 0(0.00) | 0(0.00) | 1(0.73) | 0(0.00) | 0(0.00) | 0(0.00) | 0(0.00) | 1(0.39) | 0(0.00) | 0(0.00) | 0.6237 |

| Adverse events | Hypertension group (157 assigned) | | | Diabetes group (137 assigned) | | | Combined disease group (120 assigned) | | | Healthy population (256 assigned) | | | |
|---|---|---|---|---|---|---|---|---|---|---|---|---|---|
| | Level 1 | Level 2 | Level 3 | Level 1 | Level 2 | Level 3 | Level 1 | Level 2 | Level 3 | Level 1 | Level 2 | Level 3 | *P** |
| Constipation | 0(0.00) | 0(0.00) | 0(0.00) | 0(0.00) | 0(0.00) | 0(0.00) | 1(0.83) | 0(0.00) | 0(0.00) | 0(0.00) | 0(0.00) | 0(0.00) | 0.2050 |
| Fever | 0(0.00) | 0(0.00) | 0(0.00) | 0(0.00) | 0(0.00) | 0(0.00) | 0(0.00) | 0(0.00) | 1(0.83) | 0(0.00) | 0(0.00) | 0(0.00) | 0.2050 |
| Skin mucosal | 0(0.00) | 0(0.00) | 0(0.00) | 1(0.73) | 0(0.00) | 0(0.00) | 0(0.00) | 0(0.00) | 0(0.00) | 0(0.00) | 0(0.00) | 0(0.00) | 0.2735 |
| Unsolicited event | 10(6.37) | 3(1.91) | 1(0.64) | 6(4.38) | 3(2.19) | 4(2.92) | 6(5.00) | 6(5.00) | 3(2.50) | 16(6.25) | 11(4.30) | 3(1.17) | 0.6735 |
| Musculoskeletal and connective tissue disorders | 0(0.00) | 2(1.27) | 0(0.00) | 3(2.19) | 1(0.73) | 1(0.73) | 1(0.83) | 3(2.50) | 1(0.83) | 4(1.56) | 3(1.17) | 0(0.00) | 0.4744 |
| Gastrointestinal diseases | 0(0.00) | 0(0.00) | 0(0.00) | 2(1.46) | 2(1.46) | 0(0.00) | 1(0.83) | 1(0.83) | 0(0.00) | 6(2.34) | 3(1.17) | 1(0.39) | 0.0789 |
| Systemic disease and administration site reactions | 2(1.27) | 0(0.00) | 0(0.00) | 3(2.19) | 2(1.46) | 1(0.73) | 2(1.67) | 3(2.50) | 0(0.00) | 2(0.78) | 2(0.78) | 0(0.00) | 0.1567 |
| Respiratory system, chest and mediastinal diseases | 5(3.18) | 0(0.00) | 0(0.00) | 2(1.46) | 1(0.73) | 0(0.00) | 1(0.83) | 0(0.00) | 0(0.00) | 3(1.17) | 0(0.00) | 0(0.00) | 0.3862 |
| Neurological diseases | 3(1.91) | 0(0.00) | 0(0.00) | 2(1.46) | 1(0.73) | 0(0.00) | 0(0.00) | 0(0.00) | 2(1.67) | 3(1.17) | 1(0.39) | 0(0.00) | 0.9747 |
| Metabolic and nutritional | 0(0.00) | 0(0.00) | 1(0.64) | 0(0.00) | 1(0.73) | 1(0.73) | 2(1.67) | 0(0.00) | 0(0.00) | 0(0.00) | 0(0.00) | 0(0.00) | 0.2364 |
| Kidney and urinary system | 0(0.00) | 0(0.00) | 0(0.00) | 0(0.00) | 0(0.00) | 1(0.73) | 0(0.00) | 0(0.00) | 0(0.00) | 0(0.00) | 2(0.78) | 3(1.17) | 0.1242 |
| Mental illness | 0(0.00) | 0(0.00) | 0(0.00) | 0(0.00) | 0(0.00) | 0(0.00) | 0(0.00) | 2(1.67) | 0(0.00) | 0(0.00) | 0(0.00) | 0(0.00) | 0.0270 |
| Skin and subcutaneous tissue diseases | 2(1.27) | 1(0.64) | 0(0.00) | 0(0.00) | 0(0.00) | 0(0.00) | 0(0.00) | 0(0.00) | 0(0.00) | 0(0.00) | 1(0.39) | 0(0.00) | 0.0992 |
| Infections and infectious | 0(0.00) | 0(0.00) | 0(0.00) | 0(0.00) | 0(0.00) | 1(0.73) | 1(0.83) | 0(0.00) | 1(0.83) | 0(0.00) | 0(0.00) | 0(0.00) | 0.1075 |
| Injuries, poisoning and operation complications | 1(0.64) | 0(0.00) | 0(0.00) | 0(0.00) | 0(0.00) | 0(0.00) | 0(0.00) | 0(0.00) | 0(0.00) | 0(0.00) | 0(0.00) | 2(0.78) | 0.5892 |
| Reproductive system and breast diseases | 0(0.00) | 0(0.00) | 0(0.00) | 0(0.00) | 0(0.00) | 0(0.00) | 0(0.00) | 0(0.00) | 0(0.00) | 0(0.00) | 1(0.39) | 0(0.00) | 0.6555 |
| Heart diseases | 0(0.00) | 0(0.00) | 0(0.00) | 0(0.00) | 0(0.00) | 0(0.00) | 0(0.00) | 0(0.00) | 0(0.00) | 1(0.39) | 0(0.00) | 0(0.00) | 0.6555 |

| Adverse events | Hypertension group (157 assigned) | | | Diabetes group (137 assigned) | | | Combined disease group (120 assigned) | | | Healthy population (256 assigned) | | | |
|---|---|---|---|---|---|---|---|---|---|---|---|---|---|
| | Level 1 | Level 2 | Level 3 | Level 1 | Level 2 | Level 3 | Level 1 | Level 2 | Level 3 | Level 1 | Level 2 | Level 3 | *P** |
| Blood and lymphatic system diseases | 0(0.00) | 0(0.00) | 0(0.00) | 0(0.00) | 0(0.00) | 1(0.73) | 0(0.00) | 0(0.00) | 0(0.00) | 0(0.00) | 0(0.00) | 0(0.00) | 0.2735 |

*Kruskal-Wallis test

## Table S3-2 Severity of adverse events 0-21 days after two doses of vaccine in the female

| Adverse events | Hypertension group (170 assigned) | | | Diabetes group (192 assigned) | | | Combined disease group (176 assigned) | | | Healthy population (224 assigned) | | | |
|---|---|---|---|---|---|---|---|---|---|---|---|---|---|
| | Level 1 | Level 2 | Level 3 | Level 1 | Level 2 | Level 3 | Level 1 | Level 2 | Level 3 | Level 1 | Level 2 | Level 3 | *P** |
| Total | 24(14.12) | 6(3.53) | 5(2.94) | 27(14.06) | 12(6.25) | 0(0.00) | 20(11.36) | 10(5.68) | 3(1.70) | 31(13.84) | 10(4.46) | 2(0.89) | 0.9711 |
| Solicitation event | 21(12.35) | 1(0.59) | 0(0.00) | 22(11.46) | 4(2.08) | 0(0.00) | 17(9.66) | 5(2.84) | 2(1.14) | 25(11.16) | 2(0.89) | 0(0.00) | 0.9384 |
| Local adverse events | 5(2.94) | 0(0.00) | 0(0.00) | 9(4.69) | 1(0.52) | 0(0.00) | 9(5.11) | 2(1.14) | 0(0.00) | 14(6.25) | 0(0.00) | 0(0.00) | 0.4498 |
| Soreness | 5(2.94) | 0(0.00) | 0(0.00) | 7(3.65) | 0(0.00) | 0(0.00) | 6(3.41) | 1(0.57) | 0(0.00) | 13(5.80) | 0(0.00) | 0(0.00) | 0.5210 |
| Swelling | 0(0.00) | 0(0.00) | 0(0.00) | 2(1.04) | 1(0.52) | 0(0.00) | 1(0.57) | 1(0.57) | 0(0.00) | 0(0.00) | 0(0.00) | 0(0.00) | 0.1308 |
| Pruritus | 0(0.00) | 0(0.00) | 0(0.00) | 0(0.00) | 0(0.00) | 0(0.00) | 1(0.57) | 0(0.00) | 0(0.00) | 1(0.45) | 0(0.00) | 0(0.00) | 0.6002 |
| Hardness | 0(0.00) | 0(0.00) | 0(0.00) | 0(0.00) | 0(0.00) | 0(0.00) | 1(0.57) | 0(0.00) | 0(0.00) | 0(0.00) | 0(0.00) | 0(0.00) | 0.3436 |
| Systemic adverse events | 19(11.18) | 1(0.59) | 0(0.00) | 21(10.94) | 4(2.08) | 0(0.00) | 23(13.07) | 3(1.70) | 4(2.27) | 21(9.38) | 3(1.34) | 0(0.00) | 0.2442 |
| Dizziness | 8(4.71) | 0(0.00) | 0(0.00) | 9(4.69) | 1(0.52) | 0(0.00) | 4(2.27) | 0(0.00) | 0(0.00) | 6(2.68) | 0(0.00) | 0(0.00) | 0.3326 |
| Fatigue/lethargy | 6(3.53) | 0(0.00) | 0(0.00) | 5(2.60) | 0(0.00) | 0(0.00) | 4(2.27) | 0(0.00) | 1(0.57) | 5(2.23) | 1(0.45) | 0(0.00) | 0.9535 |
| Headache | 1(0.59) | 0(0.00) | 0(0.00) | 1(0.52) | 1(0.52) | 0(0.00) | 4(2.27) | 0(0.00) | 1(0.57) | 3(1.34) | 0(0.00) | 0(0.00) | 0.3175 |
| Nausea | 0(0.00) | 0(0.00) | 0(0.00) | 1(0.52) | 0(0.00) | 0(0.00) | 3(1.70) | 0(0.00) | 0(0.00) | 1(0.45) | 0(0.00) | 0(0.00) | 0.2318 |
| Muscle pain | 1(0.59) | 0(0.00) | 0(0.00) | 2(1.04) | 0(0.00) | 0(0.00) | 0(0.00) | 0(0.00) | 0(0.00) | 2(0.89) | 0(0.00) | 0(0.00) | 0.6144 |
| Chest tightness | 0(0.00) | 0(0.00) | 0(0.00) | 1(0.52) | 0(0.00) | 0(0.00) | 3(1.70) | 0(0.00) | 0(0.00) | 1(0.45) | 0(0.00) | 0(0.00) | 0.2318 |
| Non-injection site pruritus | 1(0.59) | 0(0.00) | 0(0.00) | 0(0.00) | 0(0.00) | 0(0.00) | 1(0.57) | 0(0.00) | 0(0.00) | 1(0.45) | 1(0.45) | 0(0.00) | 0.6562 |
| Arthralgia | 0(0.00) | 0(0.00) | 0(0.00) | 0(0.00) | 1(0.52) | 0(0.00) | 2(1.14) | 1(0.57) | 0(0.00) | 0(0.00) | 0(0.00) | 0(0.00) | 0.0804 |
| Cough | 0(0.00) | 1(0.59) | 0(0.00) | 1(0.52) | 0(0.00) | 0(0.00) | 1(0.57) | 1(0.57) | 0(0.00) | 0(0.00) | 0(0.00) | 0(0.00) | 0.4839 |
| Fever | 1(0.59) | 0(0.00) | 0(0.00) | 0(0.00) | 0(0.00) | 0(0.00) | 1(0.57) | 0(0.00) | 1(0.57) | 0(0.00) | 0(0.00) | 0(0.00) | 0.2326 |
| Diarrhea | 0(0.00) | 0(0.00) | 0(0.00) | 0(0.00) | 0(0.00) | 0(0.00) | 0(0.00) | 1(0.57) | 1(0.57) | 0(0.00) | 0(0.00) | 0(0.00) | 0.0833 |

| Adverse events | Hypertension group (170 assigned) | | | Diabetes group (192 assigned) | | | Combined disease group (176 assigned) | | | Healthy population (224 assigned) | | | |
|---|---|---|---|---|---|---|---|---|---|---|---|---|---|
| | Level 1 | Level 2 | Level 3 | Level 1 | Level 2 | Level 3 | Level 1 | Level 2 | Level 3 | Level 1 | Level 2 | Level 3 | *P** |
| Vomiting | 1(0.59) | 0(0.00) | 0(0.00) | 0(0.00) | 0(0.00) | 0(0.00) | 0(0.00) | 0(0.00) | 0(0.00) | 1(0.45) | 0(0.00) | 0(0.00) | 0.5840 |
| Skin mucosal abnormalities | 0(0.00) | 0(0.00) | 0(0.00) | 1(0.52) | 1(0.52) | 0(0.00) | 0(0.00) | 0(0.00) | 0(0.00) | 0(0.00) | 0(0.00) | 0(0.00) | 0.1143 |
| Constipation | 0(0.00) | 0(0.00) | 0(0.00) | 0(0.00) | 0(0.00) | 0(0.00) | 0(0.00) | 0(0.00) | 0(0.00) | 0(0.00) | 1(0.45) | 0(0.00) | 0.4933 |
| Anorexia | 0(0.00) | 0(0.00) | 0(0.00) | 0(0.00) | 0(0.00) | 0(0.00) | 0(0.00) | 0(0.00) | 0(0.00) | 1(0.45) | 0(0.00) | 0(0.00) | 0.4933 |
| Unsolicited event | 10(5.88) | 6(3.53) | 5(2.94) | 8(4.17) | 9(4.69) | 0(0.00) | 7(3.98) | 6(3.41) | 3(1.70) | 12(5.36) | 9(4.02) | 2(0.89) | 0.6730 |
| Systemic disease and administration site reactions | 2(1.18) | 1(0.59) | 1(0.59) | 3(1.56) | 2(1.04) | 0(0.00) | 3(1.70) | 3(1.70) | 0(0.00) | 3(1.34) | 0(0.00) | 0(0.00) | 0.5863 |
| Gastrointestinal diseases | 1(0.59) | 0(0.00) | 1(0.59) | 2(1.04) | 2(1.04) | 0(0.00) | 0(0.00) | 2(1.14) | 0(0.00) | 3(1.34) | 1(0.45) | 0(0.00) | 0.8593 |
| Musculoskeletal and connective tissue disorders | 0(0.00) | 1(0.59) | 0(0.00) | 1(0.52) | 1(0.52) | 0(0.00) | 3(1.70) | 2(1.14) | 1(0.57) | 3(1.34) | 1(0.45) | 0(0.00) | 0.1853 |
| Neurological diseases | 2(1.18) | 0(0.00) | 0(0.00) | 2(1.04) | 1(0.52) | 0(0.00) | 1(0.57) | 0(0.00) | 1(0.57) | 2(0.89) | 2(0.89) | 0(0.00) | 0.9387 |
| Respiratory system, chest and mediastinal diseases | 2(1.18) | 2(1.18) | 0(0.00) | 1(0.52) | 0(0.00) | 0(0.00) | 3(1.70) | 0(0.00) | 0(0.00) | 2(0.89) | 2(0.89) | 0(0.00) | 0.5441 |
| Infections and infectious diseases | 0(0.00) | 1(0.59) | 2(1.18) | 1(0.52) | 2(1.04) | 0(0.00) | 0(0.00) | 1(0.57) | 1(0.57) | 1(0.45) | 2(0.89) | 0(0.00) | 0.9636 |
| Skin and subcutaneous tissue diseases | 0(0.00) | 0(0.00) | 1(0.59) | 1(0.52) | 0(0.00) | 0(0.00) | 0(0.00) | 0(0.00) | 0(0.00) | 1(0.45) | 2(0.89) | 0(0.00) | 0.4192 |
| Hepatobiliary diseases | 0(0.00) | 0(0.00) | 0(0.00) | 0(0.00) | 1(0.52) | 0(0.00) | 0(0.00) | 1(0.57) | 0(0.00) | 0(0.00) | 0(0.00) | 0(0.00) | 0.5415 |
| Injuries, poisoning and operation | 1(0.59) | 0(0.00) | 0(0.00) | 0(0.00) | 0(0.00) | 0(0.00) | 0(0.00) | 0(0.00) | 1(0.57) | 0(0.00) | 0(0.00) | 3(1.34) | 0.4057 |
| Eye diseases | 0(0.00) | 1(0.59) | 0(0.00) | 0(0.00) | 0(0.00) | 0(0.00) | 0(0.00) | 0(0.00) | 0(0.00) | 3(1.34) | 0(0.00) | 0(0.00) | 0.1885 |
| Kidney and urinary system diseases | 0(0.00) | 1(0.59) | 2(1.18) | 0(0.00) | 0(0.00) | 0(0.00) | 0(0.00) | 0(0.00) | 0(0.00) | 0(0.00) | 0(0.00) | 0(0.00) | 0.0149 |
| Metabolic and nutritional diseases | 0(0.00) | 0(0.00) | 1(0.59) | 0(0.00) | 1(0.52) | 0(0.00) | 0(0.00) | 0(0.00) | 0(0.00) | 0(0.00) | 0(0.00) | 0(0.00) | 0.5262 |
| Benign, malignant and unspecified tumors | 0(0.00) | 0(0.00) | 2(1.18) | 0(0.00) | 0(0.00) | 0(0.00) | 0(0.00) | 0(0.00) | 0(0.00) | 0(0.00) | 0(0.00) | 0(0.00) | 0.0727 |

| Adverse events | Hypertension group (170 assigned) | | | Diabetes group (192 assigned) | | | Combined disease group (176 assigned) | | | Healthy population (224 assigned) | | | |
|---|---|---|---|---|---|---|---|---|---|---|---|---|---|
| | Level 1 | Level 2 | Level 3 | Level 1 | Level 2 | Level 3 | Level 1 | Level 2 | Level 3 | Level 1 | Level 2 | Level 3 | *P** |
| Heart diseases | 1(0.59) | 0(0.00) | 0(0.00) | 0(0.00) | 0(0.00) | 0(0.00) | 0(0.00) | 0(0.00) | 0(0.00) | 0(0.00) | 0(0.00) | 0(0.00) | 0.3231 |
| Vascular and Lymphatic Diseases | 0(0.00) | 0(0.00) | 2(1.18) | 0(0.00) | 0(0.00) | 0(0.00) | 0(0.00) | 0(0.00) | 0(0.00) | 0(0.00) | 0(0.00) | 0(0.00) | 0.0727 |
| Inspections | 1(0.59) | 0(0.00) | 0(0.00) | 0(0.00) | 0(0.00) | 0(0.00) | 0(0.00) | 0(0.00) | 0(0.00) | 0(0.00) | 0(0.00) | 0(0.00) | 0.3231 |
| Mental illness | 0(0.00) | 0(0.00) | 0(0.00) | 0(0.00) | 0(0.00) | 0(0.00) | 0(0.00) | 0(0.00) | 0(0.00) | 1(0.45) | 0(0.00) | 0(0.00) | 0.4933 |
| Blood and lymphatic system diseases | 0(0.00) | 0(0.00) | 0(0.00) | 0(0.00) | 0(0.00) | 0(0.00) | 0(0.00) | 0(0.00) | 0(0.00) | 0(0.00) | 0(0.00) | 1(0.45) | 0.4933 |

*Kruskal-Wallis test

## Table S4-1 Adverse events 0-21 days after two doses of vaccine in people aged 60-69

| Adverse events | Hypertension group (228 assigned) | | | Diabetes group (233 assigned) | | | Combined disease group (204 assigned) | | | Healthy population (337 assigned) | | | Statistical method | Statistic | *P** |
|---|---|---|---|---|---|---|---|---|---|---|---|---|---|---|---|
| | Case times | No. of cases | Incidence rate | Case times | No. of cases | Incidence rate | Case times | No. of cases | Incidence rate | Case times | No. of cases | Incidence rate | | | |
| Total | 80 | 49 | 21.49 | 74 | 36 | 15.45 | 101 | 36 | 17.65 | 127 | 60 | 17.80 | Chi-Square Test | 2.924 | 0.4035 |
| Solicited event | 35 | 29 | 12.72 | 41 | 24 | 10.30 | 61 | 26 | 12.75 | 58 | 34 | 10.09 | Chi-Square Test | 1.611 | 0.6569 |
| Local adverse events | 11 | 11 | 4.82 | 12 | 10 | 4.29 | 13 | 10 | 4.90 | 20 | 17 | 5.04 | Chi-Square Test | 0.181 | 0.9806 |
| Soreness | 9 | 9 | 3.95 | 9 | 8 | 3.43 | 8 | 7 | 3.43 | 19 | 16 | 4.75 | Chi-Square Test | 0.860 | 0.8350 |
| Swelling | 0 | 0 | 0.00 | 3 | 3 | 1.29 | 3 | 2 | 0.98 | 0 | 0 | 0.00 | Fisher's Exact Test | - | 0.0526 |
| Pruritus | 1 | 1 | 0.44 | 0 | 0 | 0.00 | 1 | 1 | 0.49 | 1 | 1 | 0.30 | Fisher's Exact Test | - | 0.7971 |
| Rash | 1 | 1 | 0.44 | 0 | 0 | 0.00 | 0 | 0 | 0.00 | 0 | 0 | 0.00 | Fisher's Exact Test | - | 0.4311 |
| Hardness | 0 | 0 | 0.00 | 0 | 0 | 0.00 | 1 | 1 | 0.49 | 0 | 0 | 0.00 | Fisher's Exact Test | - | 0.2036 |
| Systemic adverse events | 24 | 20 | 8.77 | 29 | 19 | 8.15 | 48 | 18 | 8.82 | 38 | 22 | 6.53 | Chi-Square Test | 1.372 | 0.7121 |
| Dizziness | 7 | 6 | 2.63 | 11 | 9 | 3.86 | 8 | 7 | 3.43 | 12 | 10 | 2.97 | Chi-Square Test | 0.663 | 0.8818 |
| Fatigue/lethargy | 6 | 6 | 2.63 | 6 | 6 | 2.58 | 9 | 7 | 3.43 | 7 | 7 | 2.08 | Chi-Square Test | 0.924 | 0.8197 |
| Headache | 2 | 2 | 0.88 | 1 | 1 | 0.43 | 8 | 7 | 3.43 | 3 | 3 | 0.89 | Fisher's Exact Test | - | 0.0486 |
| Nausea | 1 | 1 | 0.44 | 3 | 3 | 1.29 | 4 | 4 | 1.96 | 1 | 1 | 0.30 | Fisher's Exact Test | - | 0.1586 |
| Chest tightness | 1 | 1 | 0.44 | 4 | 3 | 1.29 | 3 | 3 | 1.47 | 1 | 1 | 0.30 | Fisher's Exact Test | - | 0.3446 |

| Adverse events | Hypertension group (228 assigned) | | | Diabetes group (233 assigned) | | | Combined disease group (204 assigned) | | | Healthy population (337 assigned) | | | Statistical method | Statistic | *P** |
|---|---|---|---|---|---|---|---|---|---|---|---|---|---|---|---|
| | Case times | No. of cases | Incidence rate | Case times | No. of cases | Incidence rate | Case times | No. of cases | Incidence rate | Case times | No. of cases | Incidence rate | | | |
| Muscle pain | 2 | 2 | 0.88 | 2 | 2 | 0.86 | 0 | 0 | 0.00 | 4 | 3 | 0.89 | Fisher's Exact Test | - | 0.6631 |
| Cough | 2 | 2 | 0.88 | 0 | 0 | 0.00 | 5 | 5 | 2.45 | 1 | 1 | 0.30 | Fisher's Exact Test | - | 0.0163 |
| Non-injection site pruritus | 1 | 1 | 0.44 | 0 | 0 | 0.00 | 1 | 1 | 0.49 | 4 | 2 | 0.59 | Fisher's Exact Test | - | 0.7760 |
| Arthralgia | 1 | 1 | 0.44 | 0 | 0 | 0.00 | 4 | 2 | 0.98 | 1 | 1 | 0.30 | Fisher's Exact Test | - | 0.4885 |
| Fever | 0 | 0 | 0.00 | 0 | 0 | 0.00 | 3 | 3 | 1.47 | 0 | 0 | 0.00 | Fisher's Exact Test | - | 0.0083 |
| Diarrhea | 0 | 0 | 0.00 | 0 | 0 | 0.00 | 3 | 3 | 1.47 | 0 | 0 | 0.00 | Fisher's Exact Test | - | 0.0083 |
| Anorexia | 0 | 0 | 0.00 | 1 | 1 | 0.43 | 0 | 0 | 0.00 | 2 | 2 | 0.59 | Fisher's Exact Test | - | 0.7033 |
| Vomiting | 1 | 1 | 0.44 | 0 | 0 | 0.00 | 0 | 0 | 0.00 | 1 | 1 | 0.30 | Fisher's Exact Test | - | 0.8434 |
| Constipation | 0 | 0 | 0.00 | 0 | 0 | 0.00 | 0 | 0 | 0.00 | 1 | 1 | 0.30 | Fisher's Exact Test | - | 1.0000 |
| skin and mucous membrane abnormalities | 0 | 0 | 0.00 | 1 | 1 | 0.43 | 0 | 0 | 0.00 | 0 | 0 | 0.00 | Fisher's Exact Test | - | 0.6637 |
| Non-solicited event | 45 | 30 | 13.16 | 33 | 15 | 6.44 | 40 | 17 | 8.33 | 69 | 38 | 11.28 | Chi-Square Test | 7.063 | 0.0699 |
| Musculoskeletal and connective tissue disorders | 3 | 3 | 1.32 | 4 | 1 | 0.43 | 8 | 5 | 2.45 | 16 | 8 | 2.37 | Fisher's Exact Test | - | 0.2236 |
| Gastrointestinal diseases | 2 | 2 | 0.88 | 8 | 4 | 1.72 | 5 | 3 | 1.47 | 15 | 8 | 2.37 | Fisher's Exact Test | - | 0.6359 |

| Adverse events | Hypertension group (228 assigned) | | | Diabetes group (233 assigned) | | | Combined disease group (204 assigned) | | | Healthy population (337 assigned) | | | Statistical method | Statistic | *P** |
|---|---|---|---|---|---|---|---|---|---|---|---|---|---|---|---|
| | Case times | No. of cases | Incidence rate | Case times | No. of cases | Incidence rate | Case times | No. of cases | Incidence rate | Case times | No. of cases | Incidence rate | | | |
| Systemic disease and administration site reactions | 7 | 6 | 2.63 | 7 | 5 | 2.15 | 8 | 6 | 2.94 | 6 | 4 | 1.19 | Fisher's Exact Test | - | 0.4410 |
| respiratory, chest and mediastinal diseases | 9 | 8 | 3.51 | 3 | 2 | 0.86 | 2 | 2 | 0.98 | 6 | 4 | 1.19 | Fisher's Exact Test | - | 0.1219 |
| Neurological diseases | 4 | 4 | 1.75 | 3 | 3 | 1.29 | 4 | 3 | 1.47 | 5 | 4 | 1.19 | Fisher's Exact Test | - | 0.9498 |
| Infections and infectious diseases | 2 | 2 | 0.88 | 3 | 3 | 1.29 | 4 | 4 | 1.96 | 3 | 3 | 0.89 | Fisher's Exact Test | - | 0.7086 |
| Skin and subcutaneous tissue diseases | 4 | 3 | 1.32 | 1 | 1 | 0.43 | 0 | 0 | 0.00 | 4 | 4 | 1.19 | Fisher's Exact Test | - | 0.3919 |
| Metabolic and nutritional diseases | 2 | 2 | 0.88 | 2 | 2 | 0.86 | 4 | 2 | 0.98 | 0 | 0 | 0.00 | Fisher's Exact Test | - | 0.2313 |
| Kidney and urinary system diseases | 3 | 3 | 1.32 | 1 | 1 | 0.43 | 0 | 0 | 0.00 | 4 | 2 | 0.59 | Fisher's Exact Test | - | 0.4000 |
| Injury, poisoning, and operation complications | 1 | 1 | 0.44 | 0 | 0 | 0.00 | 0 | 0 | 0.00 | 5 | 3 | 0.89 | Fisher's Exact Test | - | 0.3433 |
| Mental illness | 0 | 0 | 0.00 | 0 | 0 | 0.00 | 4 | 1 | 0.49 | 1 | 1 | 0.30 | Fisher's Exact Test | - | 0.6902 |
| Eye diseases | 2 | 1 | 0.44 | 0 | 0 | 0.00 | 0 | 0 | 0.00 | 2 | 1 | 0.30 | Fisher's Exact Test | - | 0.8434 |
| heart diseases | 2 | 1 | 0.44 | 0 | 0 | 0.00 | 0 | 0 | 0.00 | 1 | 1 | 0.30 | Fisher's Exact Test | - | 0.8434 |
| Vascular and lymphatic diseases | 2 | 2 | 0.88 | 1 | 1 | 0.43 | 0 | 0 | 0.00 | 0 | 0 | 0.00 | Fisher's Exact Test | - | 0.1902 |

| Adverse events | Hypertension group (228 assigned) | | | Diabetes group (233 assigned) | | | Combined disease group (204 assigned) | | | Healthy population (337 assigned) | | | Statistical method | Statistic | *P** |
|---|---|---|---|---|---|---|---|---|---|---|---|---|---|---|---|
| | Case times | No. of cases | Incidence rate | Case times | No. of cases | Incidence rate | Case times | No. of cases | Incidence rate | Case times | No. of cases | Incidence rate | | | |
| Benign, malignant and unspecified tumors | 2 | 1 | 0.44 | 0 | 0 | 0.00 | 0 | 0 | 0.00 | 0 | 0 | 0.00 | Fisher's Exact Test | - | 0.4311 |
| Hepatobiliary diseases | 0 | 0 | 0.00 | 0 | 0 | 0.00 | 1 | 1 | 0.49 | 0 | 0 | 0.00 | Fisher's Exact Test | - | 0.2036 |
| Blood and lymphatic system diseases | 0 | 0 | 0.00 | 0 | 0 | 0.00 | 0 | 0 | 0.00 | 1 | 1 | 0.30 | Fisher's Exact Test | - | 1.0000 |

*Kruskal-Wallis test

## Table S4-2 Adverse events 0-21 days after two doses of vaccine in people aged 70 and above

| Adverse events | Hypertension group (99 assigned)) | | | Diabetes group (96 assigned)) | | | Combined disease group (92 assigned)) | | | Healthy population (143 assigned)) | | | Statistical method | Statistic | *P** |
|---|---|---|---|---|---|---|---|---|---|---|---|---|---|---|---|
| | Case times | No. of cases | Incidence rate | Case times | No. of cases | Incidence rate | Case times | No. of cases | Incidence rate | Case times | No. of cases | Incidence rate | | | |
| Total | 19 | 11 | 11.11 | 70 | 32 | 33.33 | 31 | 17 | 18.48 | 38 | 21 | 14.69 | Chi-Square Test | 18.680 | 0.0003 |
| Solicited event | 14 | 10 | 10.10 | 41 | 23 | 23.96 | 8 | 6 | 6.52 | 12 | 7 | 4.90 | Chi-Square Test | 24.427 | <0.0001 |
| Local adverse events | 2 | 2 | 2.02 | 9 | 8 | 8.33 | 2 | 2 | 2.17 | 4 | 3 | 2.10 | Fisher's Exact Test | - | 0.0699 |
| Soreness | 2 | 2 | 2.02 | 6 | 5 | 5.21 | 1 | 1 | 1.09 | 4 | 3 | 2.10 | Fisher's Exact Test | - | 0.3810 |
| Swelling | 0 | 0 | 0.00 | 2 | 2 | 2.08 | 0 | 0 | 0.00 | 0 | 0 | 0.00 | Fisher's Exact Test | - | 0.0948 |
| Pruritus | 0 | 0 | 0.00 | 1 | 1 | 1.04 | 1 | 1 | 1.09 | 0 | 0 | 0.00 | Fisher's Exact Test | - | 0.2432 |
| Systemic adverse events | 12 | 8 | 8.08 | 32 | 16 | 16.67 | 6 | 5 | 5.43 | 8 | 4 | 2.80 | Chi-Square Test | 16.431 | 0.0009 |
| Dizziness | 8 | 7 | 7.07 | 14 | 10 | 10.42 | 0 | 0 | 0.00 | 3 | 2 | 1.40 | Fisher's Exact Test | - | 0.0003 |
| Fatigue/lethargy | 2 | 1 | 1.01 | 3 | 3 | 3.13 | 1 | 1 | 1.09 | 2 | 2 | 1.40 | Fisher's Exact Test | - | 0.6869 |
| Headache | 1 | 1 | 1.01 | 6 | 6 | 6.25 | 0 | 0 | 0.00 | 0 | 0 | 0.00 | Fisher's Exact Test | - | 0.0007 |
| Non-injection site pruritus | 0 | 0 | 0.00 | 1 | 1 | 1.04 | 2 | 2 | 2.17 | 0 | 0 | 0.00 | Fisher's Exact Test | - | 0.0629 |
| Arthralgia | 0 | 0 | 0.00 | 1 | 1 | 1.04 | 1 | 1 | 1.09 | 1 | 1 | 0.70 | Fisher's Exact Test | - | 0.7977 |

| Adverse events | Hypertension group (99 assigned)) | | | Diabetes group (96 assigned)) | | | Combined disease group (92 assigned)) | | | Healthy population (143 assigned)) | | | Statistical method | Statistic | *P** |
|---|---|---|---|---|---|---|---|---|---|---|---|---|---|---|---|
| | Case times | No. of cases | Incidence rate | Case times | No. of cases | Incidence rate | Case times | No. of cases | Incidence rate | Case times | No. of cases | Incidence rate | | | |
| Cough | 0 | 0 | 0.00 | 2 | 2 | 2.08 | 0 | 0 | 0.00 | 1 | 1 | 0.70 | Fisher's Exact Test | - | 0.3612 |
| Skin mucosal abnormalities | 0 | 0 | 0.00 | 2 | 2 | 2.08 | 0 | 0 | 0.00 | 0 | 0 | 0.00 | Fisher's Exact Test | - | 0.0948 |
| Chest tightness | 0 | 0 | 0.00 | 1 | 1 | 1.04 | 1 | 1 | 1.09 | 0 | 0 | 0.00 | Fisher's Exact Test | - | 0.2432 |
| Constipation | 0 | 0 | 0.00 | 0 | 0 | 0.00 | 1 | 1 | 1.09 | 0 | 0 | 0.00 | Fisher's Exact Test | - | 0.2140 |
| Nausea | 0 | 0 | 0.00 | 1 | 1 | 1.04 | 0 | 0 | 0.00 | 0 | 0 | 0.00 | Fisher's Exact Test | - | 0.4372 |
| Fever | 1 | 1 | 1.01 | 0 | 0 | 0.00 | 0 | 0 | 0.00 | 0 | 0 | 0.00 | Fisher's Exact Test | - | 0.6674 |
| Diarrhea | 0 | 0 | 0.00 | 1 | 1 | 1.04 | 0 | 0 | 0.00 | 0 | 0 | 0.00 | Fisher's Exact Test | - | 0.4372 |
| Muscle pain | 0 | 0 | 0.00 | 0 | 0 | 0.00 | 0 | 0 | 0.00 | 1 | 1 | 0.70 | Fisher's Exact Test | - | 1.0000 |
| Non-solicited event | 5 | 5 | 5.05 | 29 | 15 | 15.63 | 23 | 14 | 15.22 | 26 | 15 | 10.49 | Chi-Square Test | 7.095 | 0.0689 |
| Musculoskeletal and connective tissue disorders | 0 | 0 | 0.00 | 7 | 5 | 5.21 | 12 | 4 | 4.35 | 3 | 2 | 1.40 | Fisher's Exact Test | - | 0.0437 |
| Systemic disease and administration site reactions | 0 | 0 | 0.00 | 9 | 5 | 5.21 | 5 | 5 | 5.43 | 4 | 2 | 1.40 | Fisher's Exact Test | - | 0.0235 |
| Gastrointestinal diseases | 0 | 0 | 0.00 | 3 | 3 | 3.13 | 2 | 1 | 1.09 | 8 | 4 | 2.80 | Fisher's Exact Test | - | 0.3069 |
| Neurological diseases | 1 | 1 | 1.01 | 3 | 2 | 2.08 | 1 | 1 | 1.09 | 4 | 4 | 2.80 | Fisher's Exact Test | - | 0.8358 |

| Adverse events | Hypertension group (99 assigned)) | | | Diabetes group (96 assigned)) | | | Combined disease group (92 assigned)) | | | Healthy population (143 assigned)) | | | Statistical method | Statistic | *P** |
|---|---|---|---|---|---|---|---|---|---|---|---|---|---|---|---|
| | Case times | No. of cases | Incidence rate | Case times | No. of cases | Incidence rate | Case times | No. of cases | Incidence rate | Case times | No. of cases | Incidence rate | | | |
| respiratory, chest and mediastinal diseases | 0 | 0 | 0.00 | 1 | 1 | 1.04 | 2 | 2 | 2.17 | 2 | 2 | 1.40 | Fisher's Exact Test | - | 0.4816 |
| Hepatobiliary diseases | 0 | 0 | 0.00 | 4 | 1 | 1.04 | 0 | 0 | 0.00 | 0 | 0 | 0.00 | Fisher's Exact Test | - | 0.4372 |
| Infections and infectious diseases | 1 | 1 | 1.01 | 1 | 1 | 1.04 | 0 | 0 | 0.00 | 0 | 0 | 0.00 | Fisher's Exact Test | - | 0.4450 |
| Injury, poisoning, and operation complications | 1 | 1 | 1.01 | 0 | 0 | 0.00 | 1 | 1 | 1.09 | 0 | 0 | 0.00 | Fisher's Exact Test | - | 0.3419 |
| Kidney and urinary system diseases | 0 | 0 | 0.00 | 0 | 0 | 0.00 | 0 | 0 | 0.00 | 2 | 2 | 1.40 | Fisher's Exact Test | - | 0.5550 |
| Reproductive system and breast diseases | 0 | 0 | 0.00 | 0 | 0 | 0.00 | 0 | 0 | 0.00 | 2 | 1 | 0.70 | Fisher's Exact Test | - | 1.0000 |
| Metabolic and nutritional diseases | 0 | 0 | 0.00 | 1 | 1 | 1.04 | 0 | 0 | 0.00 | 0 | 0 | 0.00 | Fisher's Exact Test | - | 0.4372 |
| Various inspections | 1 | 1 | 1.01 | 0 | 0 | 0.00 | 0 | 0 | 0.00 | 0 | 0 | 0.00 | Fisher's Exact Test | - | 0.6674 |
| Skin and subcutaneous tissue diseases | 1 | 1 | 1.01 | 0 | 0 | 0.00 | 0 | 0 | 0.00 | 0 | 0 | 0.00 | Fisher's Exact Test | - | 0.6674 |
| Eye diseases | 0 | 0 | 0.00 | 0 | 0 | 0.00 | 0 | 0 | 0.00 | 1 | 1 | 0.70 | Fisher's Exact Test | - | 1.0000 |

*Kruskal-Wallis test

## Table S5-1 Severity of adverse events 0-21 days after two doses of vaccine in people aged 60-69

| Adverse events | Hypertension group (228 assigned) | | | Diabetes group (233 assigned) | | | Combined disease group (204 assigned) | | | Healthy population (337 assigned) | | | *P** |
|---|---|---|---|---|---|---|---|---|---|---|---|---|---|
| | Level 1 | Level 2 | Level 3 | Level 1 | Level 2 | Level 3 | Level 1 | Level 2 | Level 3 | Level 1 | Level 2 | Level 3 | |
| Total | 27(11.84) | 2(0.88) | 1(0.44) | 19(8.15) | 3(1.29) | 1(0.43) | 21(10.29) | 5(2.45) | 3(1.47) | 31(9.20) | 5(1.48) | 0(0.00) | 0.4019 |
| Solicited event | 24(10.53) | 1(0.44) | 1(0.44) | 18(7.73) | 1(0.43) | 1(0.43) | 18(8.82) | 4(1.96) | 2(0.98) | 30(8.90) | 1(0.30) | 0(0.00) | 0.5514 |
| Local adverse events | 11(4.82) | 0(0.00) | 0(0.00) | 10(4.29) | 1(0.43) | 0(0.00) | 9(4.41) | 2(0.98) | 0(0.00) | 17(5.04) | 0(0.00) | 0(0.00) | 0.9878 |
| Soreness | 9(3.95) | 0(0.00) | 0(0.00) | 8(3.43) | 0(0.00) | 0(0.00) | 6(2.94) | 1(0.49) | 0(0.00) | 16(4.75) | 0(0.00) | 0(0.00) | 0.8384 |
| Swelling | 0(0.00) | 0(0.00) | 0(0.00) | 2(0.86) | 1(0.43) | 0(0.00) | 1(0.49) | 1(0.49) | 0(0.00) | 0(0.00) | 0(0.00) | 0(0.00) | 0.0823 |
| Pruritus | 1(0.44) | 0(0.00) | 0(0.00) | 0(0.00) | 0(0.00) | 0(0.00) | 1(0.49) | 0(0.00) | 0(0.00) | 1(0.30) | 0(0.00) | 0(0.00) | 0.7782 |
| Rash | 1(0.44) | 0(0.00) | 0(0.00) | 0(0.00) | 0(0.00) | 0(0.00) | 0(0.00) | 0(0.00) | 0(0.00) | 0(0.00) | 0(0.00) | 0(0.00) | 0.3347 |
| Hardness | 0(0.00) | 0(0.00) | 0(0.00) | 0(0.00) | 0(0.00) | 0(0.00) | 1(0.49) | 0(0.00) | 0(0.00) | 0(0.00) | 0(0.00) | 0(0.00) | 0.2712 |
| Systemic adverse events | 16(7.02) | 3(1.32) | 1(0.44) | 17(7.30) | 1(0.43) | 3(1.29) | 27(13.24) | 3(1.47) | 7(3.43) | 27(8.01) | 2(0.59) | 0(0.00) | 0.0013 |
| Dizziness | 5(2.19) | 1(0.44) | 0(0.00) | 7(3.00) | 0(0.00) | 0(0.00) | 6(2.94) | 1(0.49) | 0(0.00) | 9(2.67) | 0(0.00) | 0(0.00) | 0.9524 |
| Fatigue/lethargy | 6(2.63) | 0(0.00) | 0(0.00) | 5(2.15) | 0(0.00) | 1(0.43) | 4(1.96) | 1(0.49) | 2(0.98) | 6(1.78) | 0(0.00) | 0(0.00) | 0.6795 |
| Headache | 1(0.44) | 0(0.00) | 0(0.00) | 0(0.00) | 0(0.00) | 1(0.43) | 6(2.94) | 0(0.00) | 0(0.00) | 3(0.89) | 0(0.00) | 0(0.00) | 0.0393 |
| Nausea | 0(0.00) | 1(0.44) | 0(0.00) | 1(0.43) | 0(0.00) | 1(0.43) | 4(1.96) | 0(0.00) | 0(0.00) | 1(0.30) | 0(0.00) | 0(0.00) | 0.1789 |
| Chest tightness | 1(0.44) | 0(0.00) | 0(0.00) | 3(1.29) | 0(0.00) | 0(0.00) | 3(1.47) | 0(0.00) | 0(0.00) | 1(0.30) | 0(0.00) | 0(0.00) | 0.3466 |

| Adverse events | Hypertension group (228 assigned) | | | Diabetes group (233 assigned) | | | Combined disease group (204 assigned) | | | Healthy population (337 assigned) | | | |
|---|---|---|---|---|---|---|---|---|---|---|---|---|---|
| | Level 1 | Level 2 | Level 3 | Level 1 | Level 2 | Level 3 | Level 1 | Level 2 | Level 3 | Level 1 | Level 2 | Level 3 | *P** |
| Muscle pain | 1(0.44) | 0(0.00) | 1(0.44) | 1(0.43) | 0(0.00) | 0(0.00) | 0(0.00) | 0(0.00) | 0(0.00) | 2(0.59) | 0(0.00) | 0(0.00) | 0.6224 |
| Non-injection site pruritus | 1(0.44) | 0(0.00) | 0(0.00) | 0(0.00) | 0(0.00) | 0(0.00) | 1(0.49) | 0(0.00) | 0(0.00) | 1(0.30) | 1(0.30) | 0(0.00) | 0.7279 |
| Cough | 0(0.00) | 0(0.00) | 0(0.00) | 0(0.00) | 0(0.00) | 0(0.00) | 1(0.49) | 1(0.49) | 1(0.49) | 1(0.30) | 0(0.00) | 0(0.00) | 0.0497 |
| Fever | 0(0.00) | 0(0.00) | 0(0.00) | 0(0.00) | 0(0.00) | 0(0.00) | 1(0.49) | 0(0.00) | 2(0.98) | 0(0.00) | 0(0.00) | 0(0.00) | 0.0083 |
| Anorexia | 0(0.00) | 0(0.00) | 0(0.00) | 0(0.00) | 1(0.43) | 0(0.00) | 0(0.00) | 0(0.00) | 0(0.00) | 2(0.59) | 0(0.00) | 0(0.00) | 0.4935 |
| Diarrhea | 0(0.00) | 0(0.00) | 0(0.00) | 0(0.00) | 0(0.00) | 0(0.00) | 0(0.00) | 0(0.00) | 2(0.98) | 0(0.00) | 0(0.00) | 0(0.00) | 0.0496 |
| Arthralgia | 0(0.00) | 1(0.44) | 0(0.00) | 0(0.00) | 0(0.00) | 0(0.00) | 1(0.49) | 0(0.00) | 0(0.00) | 0(0.00) | 0(0.00) | 0(0.00) | 0.4479 |
| Vomiting | 1(0.44) | 0(0.00) | 0(0.00) | 0(0.00) | 0(0.00) | 0(0.00) | 0(0.00) | 0(0.00) | 0(0.00) | 1(0.30) | 0(0.00) | 0(0.00) | 0.6401 |
| Constipation | 0(0.00) | 0(0.00) | 0(0.00) | 0(0.00) | 0(0.00) | 0(0.00) | 0(0.00) | 0(0.00) | 0(0.00) | 0(0.00) | 1(0.30) | 0(0.00) | 0.5780 |
| Unsolicited event | 9(3.95) | 2(0.88) | 0(0.00) | 2(0.86) | 3(1.29) | 0(0.00) | 3(1.47) | 3(1.47) | 1(0.49) | 5(1.48) | 4(1.19) | 0(0.00) | 0.3882 |
| Systemic disease and administration site reactions | 3(1.32) | 0(0.00) | 0(0.00) | 1(0.43) | 1(0.43) | 0(0.00) | 1(0.49) | 3(1.47) | 0(0.00) | 1(0.30) | 1(0.30) | 0(0.00) | 0.4895 |
| respiratory, chest and mediastinal diseases | 4(1.75) | 0(0.00) | 0(0.00) | 1(0.43) | 0(0.00) | 0(0.00) | 1(0.49) | 0(0.00) | 0(0.00) | 0(0.00) | 2(0.59) | 0(0.00) | 0.3300 |
| Neurological diseases | 2(0.88) | 0(0.00) | 0(0.00) | 0(0.00) | 1(0.43) | 0(0.00) | 0(0.00) | 0(0.00) | 0(0.00) | 1(0.30) | 1(0.30) | 0(0.00) | 0.6248 |
| Mental illness | 0(0.00) | 0(0.00) | 0(0.00) | 0(0.00) | 0(0.00) | 0(0.00) | 0(0.00) | 2(0.98) | 0(0.00) | 1(0.30) | 0(0.00) | 0(0.00) | 0.2071 |

| Adverse events | Hypertension group (228 assigned) | | | Diabetes group (233 assigned) | | | Combined disease group (204 assigned) | | | Healthy population (337 assigned) | | | $P$* |
|---|---|---|---|---|---|---|---|---|---|---|---|---|---|
| | Level 1 | Level 2 | Level 3 | Level 1 | Level 2 | Level 3 | Level 1 | Level 2 | Level 3 | Level 1 | Level 2 | Level 3 | |
| Musculoskeletal and connective tissue disorders | 0(0.00) | 1(0.44) | 0(0.00) | 0(0.00) | 0(0.00) | 0(0.00) | 0(0.00) | 0(0.00) | 1(0.49) | 2(0.59) | 0(0.00) | 0(0.00) | 0.7288 |
| Gastrointestinal diseases | 0(0.00) | 0(0.00) | 0(0.00) | 0(0.00) | 2(0.86) | 0(0.00) | 0(0.00) | 1(0.49) | 0(0.00) | 1(0.30) | 0(0.00) | 0(0.00) | 0.5154 |
| Metabolic and nutritional diseases | 0(0.00) | 0(0.00) | 0(0.00) | 0(0.00) | 1(0.43) | 0(0.00) | 1(0.49) | 0(0.00) | 0(0.00) | 0(0.00) | 0(0.00) | 0(0.00) | 0.4561 |
| heart diseases | 1(0.44) | 0(0.00) | 0(0.00) | 0(0.00) | 0(0.00) | 0(0.00) | 0(0.00) | 0(0.00) | 0(0.00) | 0(0.00) | 0(0.00) | 0(0.00) | 0.3347 |
| Eye diseases | 0(0.00) | 1(0.44) | 0(0.00) | 0(0.00) | 0(0.00) | 0(0.00) | 0(0.00) | 0(0.00) | 0(0.00) | 0(0.00) | 0(0.00) | 0(0.00) | 0.3347 |
| Infections and infectious diseases | 0(0.00) | 0(0.00) | 0(0.00) | 0(0.00) | 1(0.43) | 0(0.00) | 0(0.00) | 0(0.00) | 0(0.00) | 0(0.00) | 0(0.00) | 0(0.00) | 0.3476 |
| Skin and subcutaneous tissue diseases | 0(0.00) | 0(0.00) | 0(0.00) | 0(0.00) | 0(0.00) | 0(0.00) | 0(0.00) | 0(0.00) | 0(0.00) | 0(0.00) | 1(0.30) | 0(0.00) | 0.5780 |
| Kidney and urinary system diseases | 0(0.00) | 0(0.00) | 0(0.00) | 0(0.00) | 0(0.00) | 0(0.00) | 0(0.00) | 0(0.00) | 0(0.00) | 1(0.30) | 0(0.00) | 0(0.00) | 0.5780 |

*Kruskal-Wallis test

## Table S5-2 Severity of adverse events 0-21 days after two doses of vaccine in people aged 70 and above

| Adverse events | Hypertension group (99 assigned) | | | Diabetes group (96 assigned) | | | Combined disease group (92 assigned) | | | Healthy population (143 assigned) | | | *P** |
|---|---|---|---|---|---|---|---|---|---|---|---|---|---|
| | Level 1 | Level 2 | Level 3 | Level 1 | Level 2 | Level 3 | Level 1 | Level 2 | Level 3 | Level 1 | Level 2 | Level 3 | |
| Total | 9(9.09) | 2(2.02) | 0(0.00) | 19(19.79) | 12(12.50) | 1(1.04) | 10(10.87) | 5(5.43) | 2(2.17) | 13(9.09) | 7(4.90) | 1(0.70) | 0.0003 |
| Solicited event | 10(10.10) | 0(0.00) | 0(0.00) | 16(16.67) | 7(7.29) | 0(0.00) | 4(4.35) | 2(2.17) | 0(0.00) | 6(4.20) | 1(0.70) | 0(0.00) | <0.0001 |
| Local adverse events | 2(2.02) | 0(0.00) | 0(0.00) | 8(8.33) | 0(0.00) | 0(0.00) | 2(2.17) | 0(0.00) | 0(0.00) | 3(2.10) | 0(0.00) | 0(0.00) | 0.0351 |
| Soreness | 2(2.02) | 0(0.00) | 0(0.00) | 5(5.21) | 0(0.00) | 0(0.00) | 1(1.09) | 0(0.00) | 0(0.00) | 3(2.10) | 0(0.00) | 0(0.00) | 0.2920 |
| Swelling | 0(0.00) | 0(0.00) | 0(0.00) | 2(2.08) | 0(0.00) | 0(0.00) | 0(0.00) | 0(0.00) | 0(0.00) | 0(0.00) | 0(0.00) | 0(0.00) | 0.0727 |
| Pruritus | 0(0.00) | 0(0.00) | 0(0.00) | 1(1.04) | 0(0.00) | 0(0.00) | 1(1.09) | 0(0.00) | 0(0.00) | 0(0.00) | 0(0.00) | 0(0.00) | 0.4606 |
| Systemic adverse events | 10(10.10) | 0(0.00) | 0(0.00) | 19(19.79) | 9(9.38) | 0(0.00) | 4(4.35) | 2(2.17) | 0(0.00) | 6(4.20) | 1(0.70) | 0(0.00) | <0.0001 |
| Dizziness | 7(7.07) | 0(0.00) | 0(0.00) | 8(8.33) | 2(2.08) | 0(0.00) | 0(0.00) | 0(0.00) | 0(0.00) | 2(1.40) | 0(0.00) | 0(0.00) | 0.0006 |
| Fatigue/lethargy | 1(1.01) | 0(0.00) | 0(0.00) | 2(2.08) | 1(1.04) | 0(0.00) | 1(1.09) | 0(0.00) | 0(0.00) | 2(1.40) | 0(0.00) | 0(0.00) | 0.6122 |
| Headache | 1(1.01) | 0(0.00) | 0(0.00) | 4(4.17) | 2(2.08) | 0(0.00) | 0(0.00) | 0(0.00) | 0(0.00) | 0(0.00) | 0(0.00) | 0(0.00) | 0.0007 |
| Non-injection site pruritus | 0(0.00) | 0(0.00) | 0(0.00) | 1(1.04) | 0(0.00) | 0(0.00) | 1(1.09) | 1(1.09) | 0(0.00) | 0(0.00) | 0(0.00) | 0(0.00) | 0.1907 |
| Arthralgia | 0(0.00) | 0(0.00) | 0(0.00) | 0(0.00) | 1(1.04) | 0(0.00) | 0(0.00) | 1(1.09) | 0(0.00) | 1(0.70) | 0(0.00) | 0(0.00) | 0.7860 |
| Cough | 0(0.00) | 0(0.00) | 0(0.00) | 1(1.04) | 1(1.04) | 0(0.00) | 0(0.00) | 0(0.00) | 0(0.00) | 0(0.00) | 1(0.70) | 0(0.00) | 0.2632 |
| Skin mucosal abnormalities | 0(0.00) | 0(0.00) | 0(0.00) | 1(1.04) | 1(1.04) | 0(0.00) | 0(0.00) | 0(0.00) | 0(0.00) | 0(0.00) | 0(0.00) | 0(0.00) | 0.0727 |

| | Hypertension group (99 assigned) | | | Diabetes group (96 assigned) | | | Combined disease group (92 assigned) | | | Healthy population (143 assigned) | | | |
|---|---|---|---|---|---|---|---|---|---|---|---|---|---|
| Adverse events | Level 1 | Level 2 | Level 3 | Level 1 | Level 2 | Level 3 | Level 1 | Level 2 | Level 3 | Level 1 | Level 2 | Level 3 | *P** |
| Chest tightness | 0(0.00) | 0(0.00) | 0(0.00) | 1(1.04) | 0(0.00) | 0(0.00) | 1(1.09) | 0(0.00) | 0(0.00) | 0(0.00) | 0(0.00) | 0(0.00) | 0.4606 |
| Constipation | 0(0.00) | 0(0.00) | 0(0.00) | 0(0.00) | 0(0.00) | 0(0.00) | 1(1.09) | 0(0.00) | 0(0.00) | 0(0.00) | 0(0.00) | 0(0.00) | 0.2989 |
| Nausea | 0(0.00) | 0(0.00) | 0(0.00) | 1(1.04) | 0(0.00) | 0(0.00) | 0(0.00) | 0(0.00) | 0(0.00) | 0(0.00) | 0(0.00) | 0(0.00) | 0.3235 |
| Fever | 1(1.01) | 0(0.00) | 0(0.00) | 0(0.00) | 0(0.00) | 0(0.00) | 0(0.00) | 0(0.00) | 0(0.00) | 0(0.00) | 0(0.00) | 0(0.00) | 0.3416 |
| Diarrhea | 0(0.00) | 0(0.00) | 0(0.00) | 0(0.00) | 1(1.04) | 0(0.00) | 0(0.00) | 0(0.00) | 0(0.00) | 0(0.00) | 0(0.00) | 0(0.00) | 0.3235 |
| Muscle pain | 0(0.00) | 0(0.00) | 0(0.00) | 0(0.00) | 0(0.00) | 0(0.00) | 0(0.00) | 0(0.00) | 0(0.00) | 1(0.70) | 0(0.00) | 0(0.00) | 0.5710 |
| Non-solicited event | 3(3.03) | 2(2.02) | 0(0.00) | 7(7.29) | 7(7.29) | 1(1.04) | 7(7.61) | 5(5.43) | 2(2.17) | 8(5.59) | 6(4.20) | 1(0.70) | 0.0646 |
| Musculoskeletal and connective tissue disorders | 0(0.00) | 0(0.00) | 0(0.00) | 4(4.17) | 1(1.04) | 1(1.04) | 3(3.26) | 3(3.26) | 0(0.00) | 1(0.70) | 1(0.70) | 0(0.00) | 0.0134 |
| Systemic disease and administration site reactions | 0(0.00) | 0(0.00) | 0(0.00) | 2(2.08) | 3(3.13) | 0(0.00) | 3(3.26) | 2(2.17) | 0(0.00) | 1(0.70) | 1(0.70) | 0(0.00) | 0.0406 |
| Gastrointestinal diseases | 0(0.00) | 0(0.00) | 0(0.00) | 3(3.13) | 0(0.00) | 0(0.00) | 0(0.00) | 1(1.09) | 0(0.00) | 2(1.40) | 3(2.10) | 0(0.00) | 0.2198 |
| Neurological diseases | 1(1.01) | 0(0.00) | 0(0.00) | 3(3.13) | 0(0.00) | 0(0.00) | 0(0.00) | 0(0.00) | 1(1.09) | 3(2.10) | 1(0.70) | 0(0.00) | 0.6075 |
| Respiratory, chest and mediastinal diseases | 0(0.00) | 0(0.00) | 0(0.00) | 0(0.00) | 1(1.04) | 0(0.00) | 2(2.17) | 0(0.00) | 0(0.00) | 2(1.40) | 0(0.00) | 0(0.00) | 0.5625 |
| Hepatobiliary diseases | 0(0.00) | 0(0.00) | 0(0.00) | 0(0.00) | 1(1.04) | 0(0.00) | 0(0.00) | 0(0.00) | 0(0.00) | 0(0.00) | 0(0.00) | 0(0.00) | 0.3235 |

| Adverse events | Hypertension group (99 assigned) | | | Diabetes group (96 assigned) | | | Combined disease group (92 assigned) | | | Healthy population (143 assigned) | | | *P** |
|---|---|---|---|---|---|---|---|---|---|---|---|---|---|
| | Level 1 | Level 2 | Level 3 | Level 1 | Level 2 | Level 3 | Level 1 | Level 2 | Level 3 | Level 1 | Level 2 | Level 3 | |
| Infections and infectious diseases | 0(0.00) | 1(1.01) | 0(0.00) | 0(0.00) | 1(1.04) | 0(0.00) | 0(0.00) | 0(0.00) | 0(0.00) | 0(0.00) | 0(0.00) | 0(0.00) | 0.4905 |
| Injury, poisoning, and operation complications | 1(1.01) | 0(0.00) | 0(0.00) | 0(0.00) | 0(0.00) | 0(0.00) | 0(0.00) | 0(0.00) | 1(1.09) | 0(0.00) | 0(0.00) | 0(0.00) | 0.4725 |
| Kidney and urinary system diseases | 0(0.00) | 0(0.00) | 0(0.00) | 0(0.00) | 0(0.00) | 0(0.00) | 0(0.00) | 0(0.00) | 0(0.00) | 0(0.00) | 1(0.70) | 1(0.70) | 0.2590 |
| Reproductive system and breast diseases | 0(0.00) | 0(0.00) | 0(0.00) | 0(0.00) | 0(0.00) | 0(0.00) | 0(0.00) | 0(0.00) | 0(0.00) | 0(0.00) | 1(0.70) | 0(0.00) | 0.5710 |
| Metabolic and nutritional diseases | 0(0.00) | 0(0.00) | 0(0.00) | 0(0.00) | 1(1.04) | 0(0.00) | 0(0.00) | 0(0.00) | 0(0.00) | 0(0.00) | 0(0.00) | 0(0.00) | 0.3235 |
| Various inspections | 1(1.01) | 0(0.00) | 0(0.00) | 0(0.00) | 0(0.00) | 0(0.00) | 0(0.00) | 0(0.00) | 0(0.00) | 0(0.00) | 0(0.00) | 0(0.00) | 0.3416 |
| Skin and subcutaneous tissue diseases | 0(0.00) | 1(1.01) | 0(0.00) | 0(0.00) | 0(0.00) | 0(0.00) | 0(0.00) | 0(0.00) | 0(0.00) | 0(0.00) | 0(0.00) | 0(0.00) | 0.3416 |
| Eye diseases | 0(0.00) | 0(0.00) | 0(0.00) | 0(0.00) | 0(0.00) | 0(0.00) | 0(0.00) | 0(0.00) | 0(0.00) | 1(0.70) | 0(0.00) | 0(0.00) | 0.5710 |

**Kruskal-Wallis test
